# Supplementary material for: A process-based assessment of landscape change and salmon habitat losses in the Chehalis River basin, USA
Source: PLoS One. 2021 Nov 2;16(11):e0258251. doi: 10.1371/journal.pone.0258251 (PMC8562855; doi:10.1371/journal.pone.0258251)

S1 Figure. Habitat distributions by species. Distribution of spawning and rearing habitat for each of the four salmon runs in the Chehalis River basin (http://geo.wa.gov/datasets/4ed1382bad264555b018cc8c934f1c01_0). Distributions represent potential distribution, including habitat above man-made migration barriers.

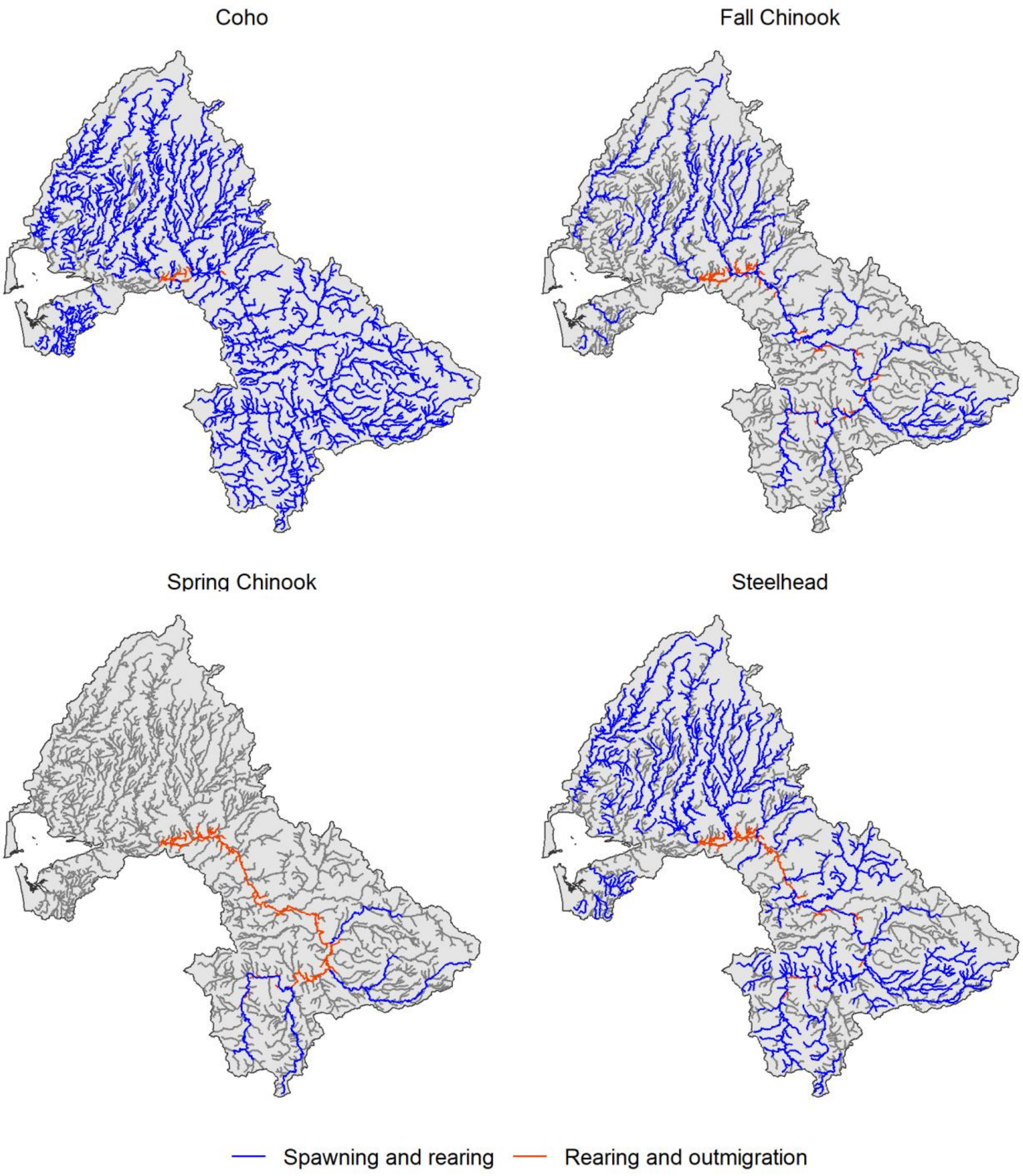

Supplement: S1 Fig — Distribution of spawning and rearing habitat for each of the four salmon runs in the Chehalis River basin (http://geo.wa.gov/datasets/4ed1382bad264555b018cc8c934f1c01_0). Distributions represent potential distribution, including habitat above man-made migration barriers. (PDF) [file pone.0258251.s001.pdf]
