# Supplementary material for: A process-based assessment of landscape change and salmon habitat losses in the Chehalis River basin, USA
Source: PLoS One. 2021 Nov 2;16(11):e0258251. doi: 10.1371/journal.pone.0258251 (PMC8562855; doi:10.1371/journal.pone.0258251)

S2 Figure. Edge unit widths. Relationship of bar edge, natural bank edge (N bank edge) and armored bank edge (M bank edge) unit widths to total wetted width at 119 transects in the Chehalis River basin. To extrapolate edge unit widths across the Chehalis River basin, we used the regression equations to estimate edge unit widths for all edge habitats based on the mean estimated wetted width of each reach. For bar units and bank units without corresponding hydromodified banks, the historical unit area was considered the same as the current unit area. For modified bank units, area of modified banks represented the current bank habitat, and area of corresponding natural bank (the copy) represented the historical bank habitat.

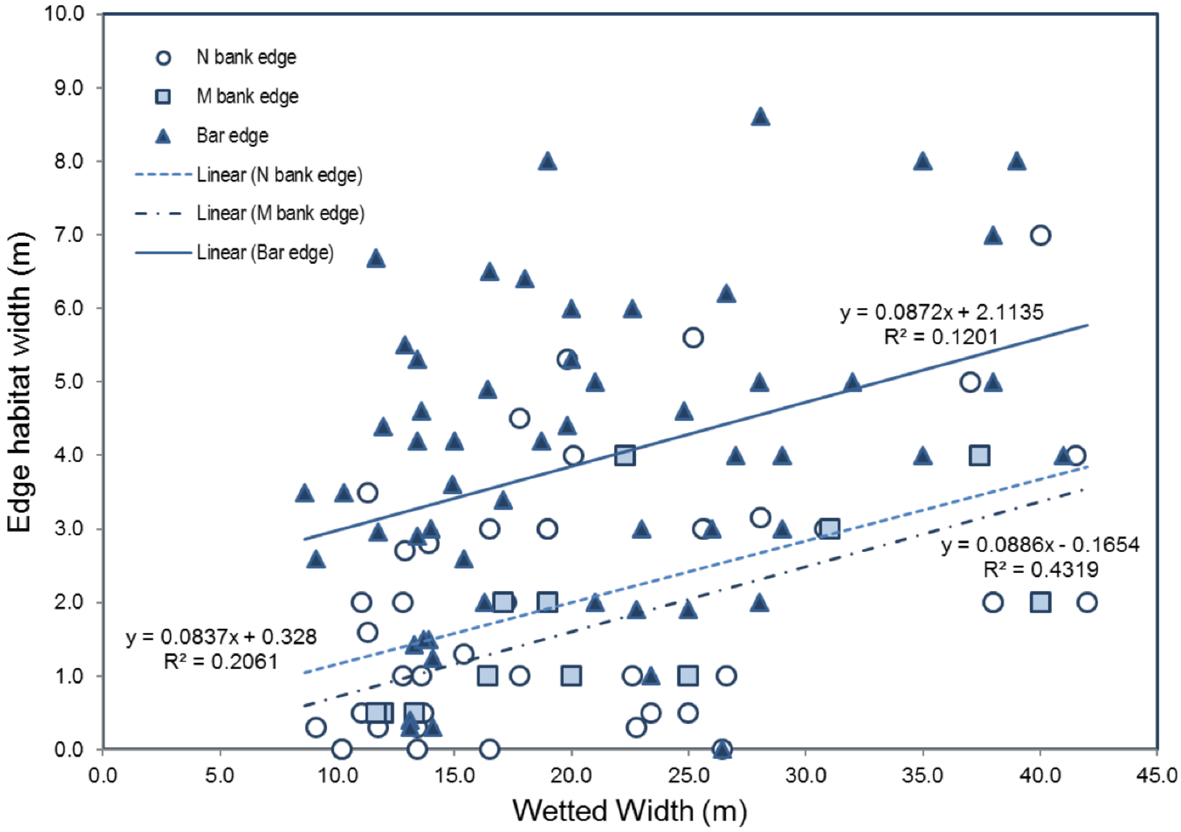

Supplement: S2 Fig — Relationship of bar edge, natural bank edge (N bank edge) and armored bank edge (M bank edge) unit widths to total wetted width at 119 transects in the Chehalis River basin. To extrapolate edge unit widths across the Chehalis River basin, we used the regression equations to estimate edge unit widths for all edge habitats based on the mean estimated wetted width of each reach. For bar units and bank units without corresponding hydromodified banks, the historical unit area was considered the same as the current unit area. For modified bank units, area of modified banks represented the current bank habitat, and area of corresponding natural bank (the copy) represented the historical bank habitat. (PDF) [file pone.0258251.s002.pdf]
