# Supplementary material for: A process-based assessment of landscape change and salmon habitat losses in the Chehalis River basin, USA
Source: PLoS One. 2021 Nov 2;16(11):e0258251. doi: 10.1371/journal.pone.0258251 (PMC8562855; doi:10.1371/journal.pone.0258251)

S3 Figure. Lidar tree heights by size class. Box and whiskers plots of lidar tree heights in each size class identified on aerial photography (bar is the median, box represents the 25th to 75th percentiles, whiskers represent the 10th and 90th percentiles, and circles are "outliers"). Each point represents one point at which an observer classified a tree size class on aerial photography and tree height in the lidar data set. There were two observers, and for each observer there were a total of 232 point measurements at 116 sites (one point on each side of the stream at each site), so there were a total of 464 points. Sample sizes for each size class are: Tall = 29, Medium = 307, Short = 104, and No veg = 24.

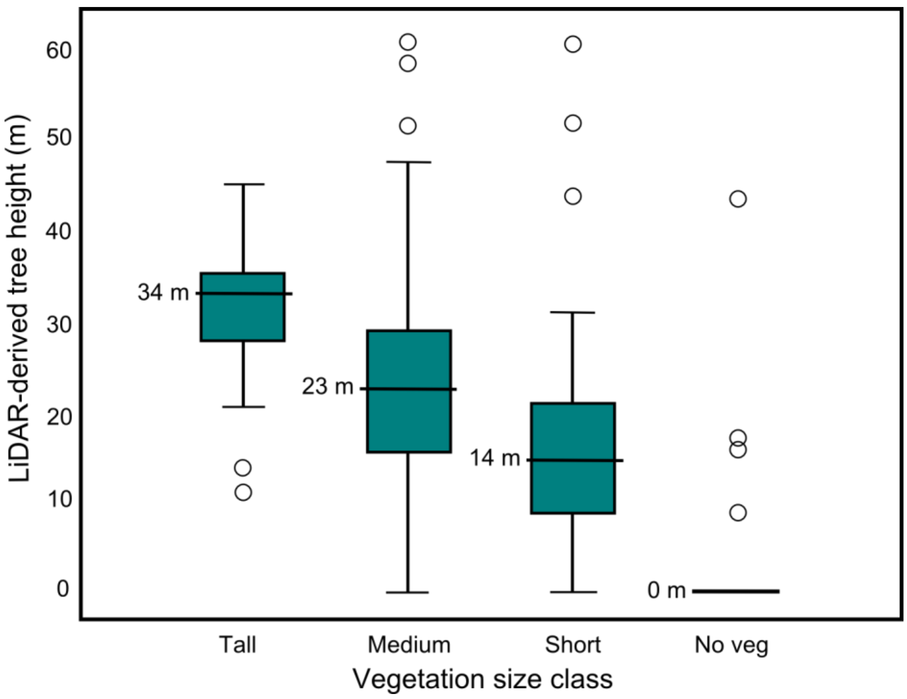

Supplement: S3 Fig — Box and whiskers plots of lidar tree heights in each size class identified on aerial photography (bar is the median, box represents the 25th to 75th percentiles, whiskers represent the 10th and 90th percentiles, and circles are “outliers”). Each point represents one point at which an observer classified a tree size class on aerial photography and tree height in the lidar data set. There were two observers, and for each observer there were a total of 232 point measurements at 116 sites (one point on each side of the stream at each site), so there were a total of 464 points. Sample sizes for each size class are: Tall = 29, Medium = 307, Short = 104, and No veg = 24. (PDF) [file pone.0258251.s003.pdf]
