# Supplementary material for: A process-based assessment of landscape change and salmon habitat losses in the Chehalis River basin, USA
Source: PLoS One. 2021 Nov 2;16(11):e0258251. doi: 10.1371/journal.pone.0258251 (PMC8562855; doi:10.1371/journal.pone.0258251)
Supplement: S4 Fig — For riparian reference conditions (e.g., the natural potential tree height), we stratified the basin into non-floodplain channels with stable riparian landforms (terraces or hill slopes, upper panel), and floodplain channels with varying rates of lateral channel migration and floodplain turnover. Channels with narrow or no floodplain are typically dominated by upland forest types in western Washington [1, 2]. Floodplain channels have floodplains >4 times the width of the main channel, and multiple side channels may flow across the floodplain [3, 4]. Because these channels constantly erode floodplain surfaces at one location and create new ones at other locations, the riparian forest consists of many small stands of varying ages and species compositions [5]. (PDF) [file pone.0258251.s004.pdf]

S4 Figure. Geomorphic settings for riparian reference conditions. For riparian reference conditions (e.g., the natural potential tree height), we stratified the basin into non-floodplain channels with stable riparian landforms (terraces or hill slopes, upper panel), and floodplain channels with varying rates of lateral channel migration and floodplain turnover. Channels with narrow or no floodplain are typically dominated by upland forest types in western Washington [1,2]. Floodplain channels have floodplains >4 times the width of the main channel, and multiple side channels may flow across the floodplain [3,4]. Because these channels constantly erode floodplain surfaces at one location and create new ones at other locations, the riparian forest consists of many small stands of varying ages and species compositions [5].

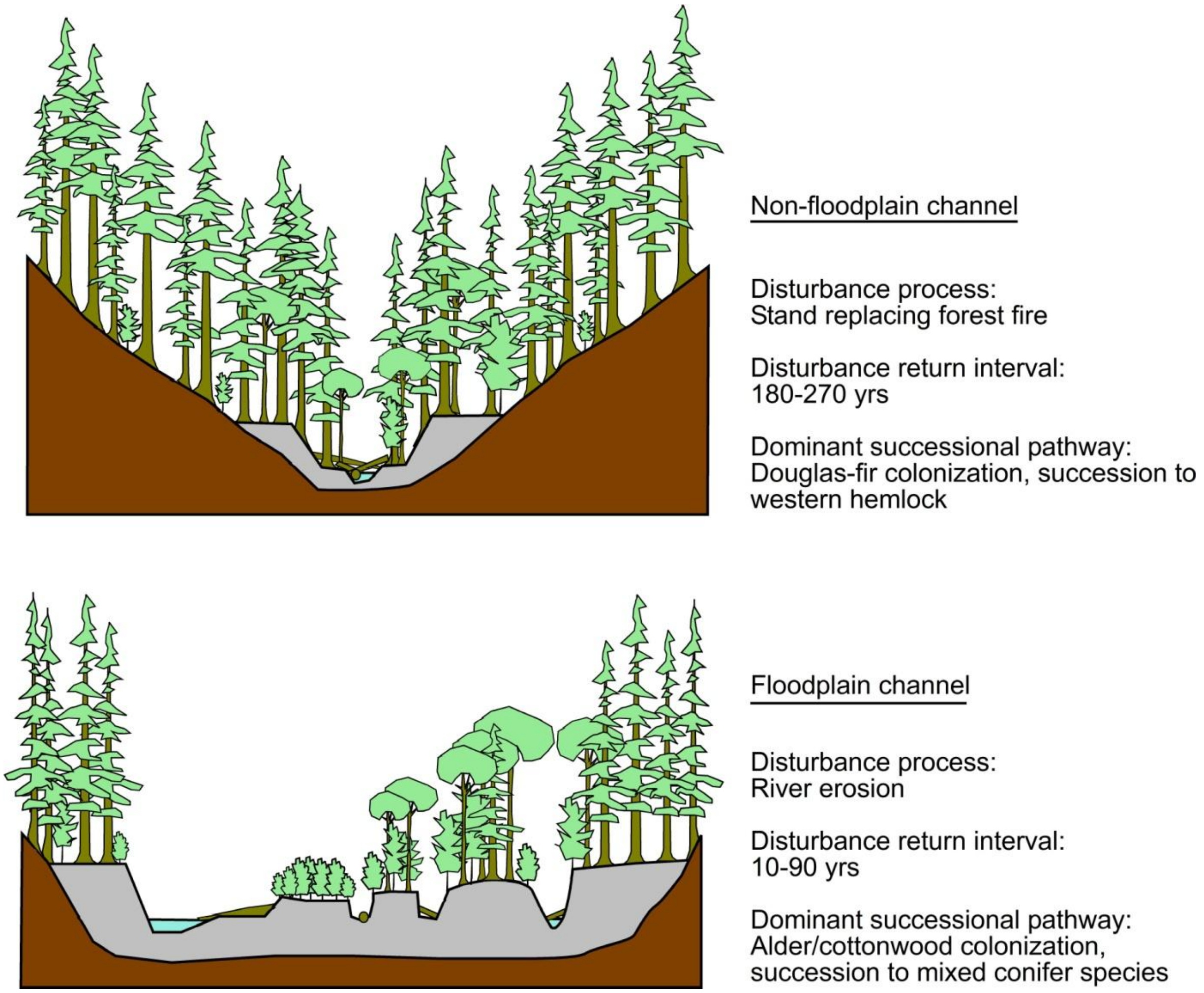

References

1. Beechie TJ, Pess G, Kennard P, Bilby RE, Bolton S. Modeling recovery rates and pathways for woody debris recruitment in northwestern Washington streams. North American Journal of Fisheries Management. 2000;20: 436–452. doi:10.1577/1548-8675(2000)020<0436:MRRAPF>2.3.CO;2

2. Rot BW, Naiman RJ, Bilby RE. Stream channel configuration, landform, and riparian forest structure in the Cascade Mountains, Washington. Can J Fish Aquat Sci. 2000;57: 699–707. doi:10.1139/f00-002

3. Latterell JJ, Scott Bechtold J, O'keefe TC, Van Pelt R, Naiman RJ. Dynamic patch mosaics and channel movement in an unconfined river valley of the Olympic Mountains. Freshwater Biology. 2006;51: 523–544. doi:10.1111/j.1365-2427.2006.01513.x

4. Beechie TJ, Liermann M, Pollock MM, Baker S, Davies J. Channel pattern and river-floodplain dynamics in forested mountain river systems. Geomorphology. 2006;78: 124–141. doi:10.1016/j.geomorph.2006.01.030

5. Naiman RJ, Bechtold JS, Beechie TJ, Latterell JJ, Van Pelt R. A process-based view of floodplain forest patterns in coastal river valleys of the Pacific Northwest. Ecosystems. 2010;13: 1–31. doi:10.1007/s10021-009-9298-5
