# Supplementary material for: A process-based assessment of landscape change and salmon habitat losses in the Chehalis River basin, USA
Source: PLoS One. 2021 Nov 2;16(11):e0258251. doi: 10.1371/journal.pone.0258251 (PMC8562855; doi:10.1371/journal.pone.0258251)
Supplement: S5 Fig — Non-floodplain channels have longer disturbance return intervals relative to floodplain channels, and conifer-dominated forests. The left panels show cumulative stand age distributions under modeled fire return intervals and forest management for non-floodplain channels [1] and erosion return intervals for floodplain channels [2]. Right panels show species compositions by land form for floodplain and non-floodplain channels [3, 4]. Natural potential tree heights used in the model were 52 m for non-floodplain channels and 30.5 m for floodplain channels based on tree heights for Douglas-fir for non-floodplain channels and red alder for floodplain channels [5]. (PDF) [file pone.0258251.s005.pdf]

S5 Figure. Reference conditions for non-floodplain and floodplain channels. Non-floodplain channels have longer disturbance return intervals relative to floodplain channels, and conifer-dominated forests. The left panels show cumulative stand age distributions under modeled fire return intervals and forest management for non-floodplain channels [1] and erosion return intervals for floodplain channels [2]. Right panels show species compositions by land form for floodplain and non-floodplain channels [3,4]. Natural potential tree heights used in the model were 52 m for non-floodplain channels and 30.5 m for floodplain channels based on tree heights for Douglas-fir for non-floodplain channels and red alder for floodplain channels [5].

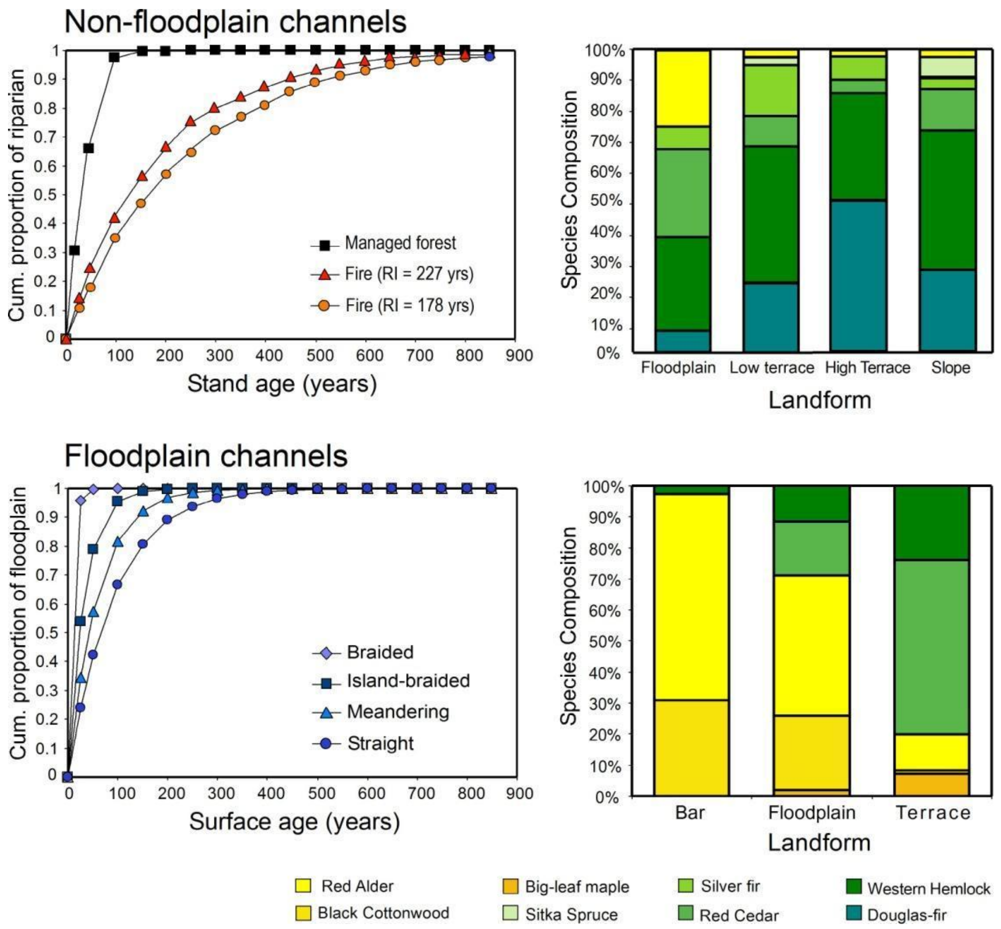

References

1. Beechie TJ. Rates and pathways of recovery for sediment supply and woody debris recruitment in northwestern Washington streams, and implications for salmonid habitat restoration. Ph.D. Dissertation, University of Washington. 1998.

2. Beechie TJ, Liermann M, Pollock MM, Baker S, Davies J. Channel pattern and river-floodplain dynamics in forested mountain river systems. Geomorphology. 2006;78: 124–141. doi:10.1016/j.geomorph.2006.01.030

3. Rot BW, Naiman RJ, Bilby RE. Stream channel configuration, landform, and riparian forest structure in the Cascade Mountains, Washington. Can J Fish Aquat Sci. 2000;57: 699–707. doi:10.1139/f00-002

4. Van Pelt R, O'Keefe TC, Latterell JJ, Naiman RJ. Riparian forest stand development along the Queets River in Olympic National Park, Washington. Ecological Monographs. 2006;76: 277–298. doi:10.1890/05-0753

5. Beechie TJ, Pess G, Kennard P, Bilby RE, Bolton S. Modeling recovery rates and pathways for woody debris recruitment in northwestern Washington streams. North American Journal of Fisheries Management. 2000;20: 436–452. doi:10.1577/1548-8675(2000)020<0436:MRRAPF>2.3.CO;2
