# Supplementary material for: A process-based assessment of landscape change and salmon habitat losses in the Chehalis River basin, USA
Source: PLoS One. 2021 Nov 2;16(11):e0258251. doi: 10.1371/journal.pone.0258251 (PMC8562855; doi:10.1371/journal.pone.0258251)
Supplement: S6 Fig — Illustration of canopy opening angle (θ) and the parameters used to calculate it [1]. Left bank tree height and right bank tree height are zL and zR, respectively, and W is bankfull channel width. The equation for calculating the canopy opening angle is: θ=(90−tan−1(zLW2))+(90−tan−1(zRW2)) The inverse tangent functions are subtracted from 90°, so a channel with complete canopy closure will have θ = 0° and a channel with no vegetation on either bank will have θ = 180°. (PDF) [file pone.0258251.s006.pdf]

S6 Figure. Canopy opening angle diagram. Illustration of canopy opening angle (θ) and the parameters used to calculate it [1]. Left bank tree height and right bank tree height are z_L and z_R, respectively, and W is bankfull channel width. The equation for calculating the canopy opening angle is:

$$\theta = \left(90 - tan^{-1}\left(\frac{z_L}{\frac{W}{2}}\right)\right) + \left(90 - tan^{-1}\left(\frac{z_R}{\frac{W}{2}}\right)\right)$$

The inverse tangent functions are subtracted from 90°, so a channel with complete canopy closure will have θ = 0° and a channel with no vegetation on either bank will have θ = 180°.

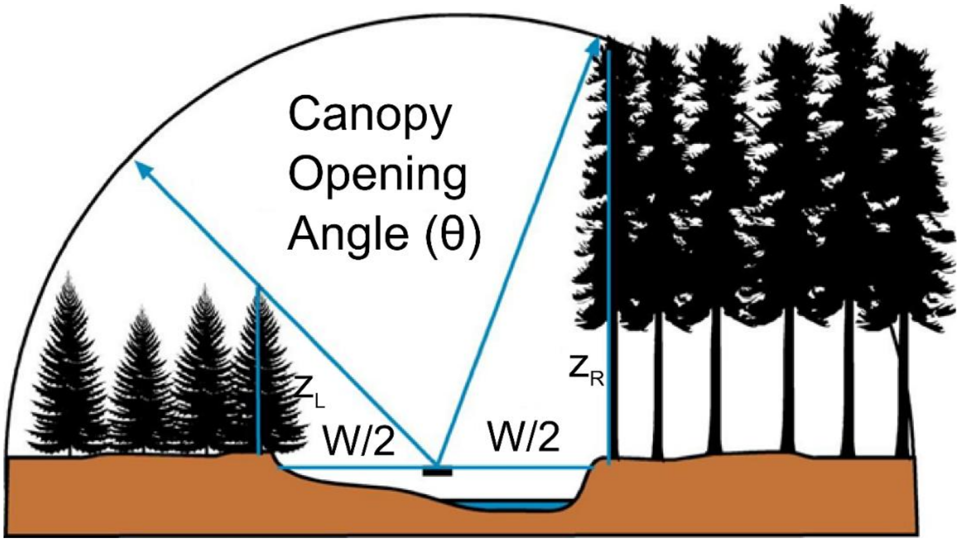

References

1. Seixas GB, Beechie TJ, Fogel C, Kiffney PM. Historical and future stream temperature change predicted by a lidar-based assessment of riparian condition and channel width. Journal of the American Water Resources Association. 2018;54: 974–991. doi:10.1111/1752-1688.12655
