# Supplementary material for: A process-based assessment of landscape change and salmon habitat losses in the Chehalis River basin, USA
Source: PLoS One. 2021 Nov 2;16(11):e0258251. doi: 10.1371/journal.pone.0258251 (PMC8562855; doi:10.1371/journal.pone.0258251)
Supplement: S7 Fig — (A) Comparison of fine sediment values and road density from the Chehalis (circles) [1] to published values of fine sediment plotted against road density for the Queets and Clearwater Rivers on the Olympic Peninsula (triangles). The line represents the linear model of percent fines to road density [2]. Note that samples from Scatter Creek and Mima Creek tributary (Mima trib) have particularly high percent fines at relatively low road density. (B) Percent fines plotted against the shear stress index (slope × bankfull width). Below the threshold of 0.05 (dotted line), the average fine sediment of the six sites is 27.6% fines. We estimated road density using the Flow Accumulation tool, which counts all cells upstream of any given cell, as well as the number of road cells upstream of that cell. An accuracy test showed that this more efficient method produced road area consistently 1.3 times higher than a more accurate but computationally intensive line-derived road area. Therefore, we used a correction factor of 0.767 on all raster road areas to increase accuracy. (PDF) [file pone.0258251.s007.pdf]

S7 Figure. Fine sediment analysis. (A) Comparison of fine sediment values and road density from the Chehalis (circles) [1] to published values of fine sediment plotted against road density for the Queets and Clearwater Rivers on the Olympic Peninsula (triangles). The line represents the linear model of percent fines to road density [2]. Note that samples from Scatter Creek and Mima Creek tributary (Mima trib) have particularly high percent fines at relatively low road density. (B) Percent fines plotted against the shear stress index (*slope* × *bankfull width*). Below the threshold of 0.05 (dotted line), the average fine sediment of the six sites is 27.6% fines. We estimated road density using the Flow Accumulation tool, which counts all cells upstream of any given cell, as well as the number of road cells upstream of that cell. An accuracy test showed that this more efficient method produced road area consistently 1.3 times higher than a more accurate but computationally intensive line-derived road area. Therefore, we used a correction factor of 0.767 on all raster road areas to increase accuracy.

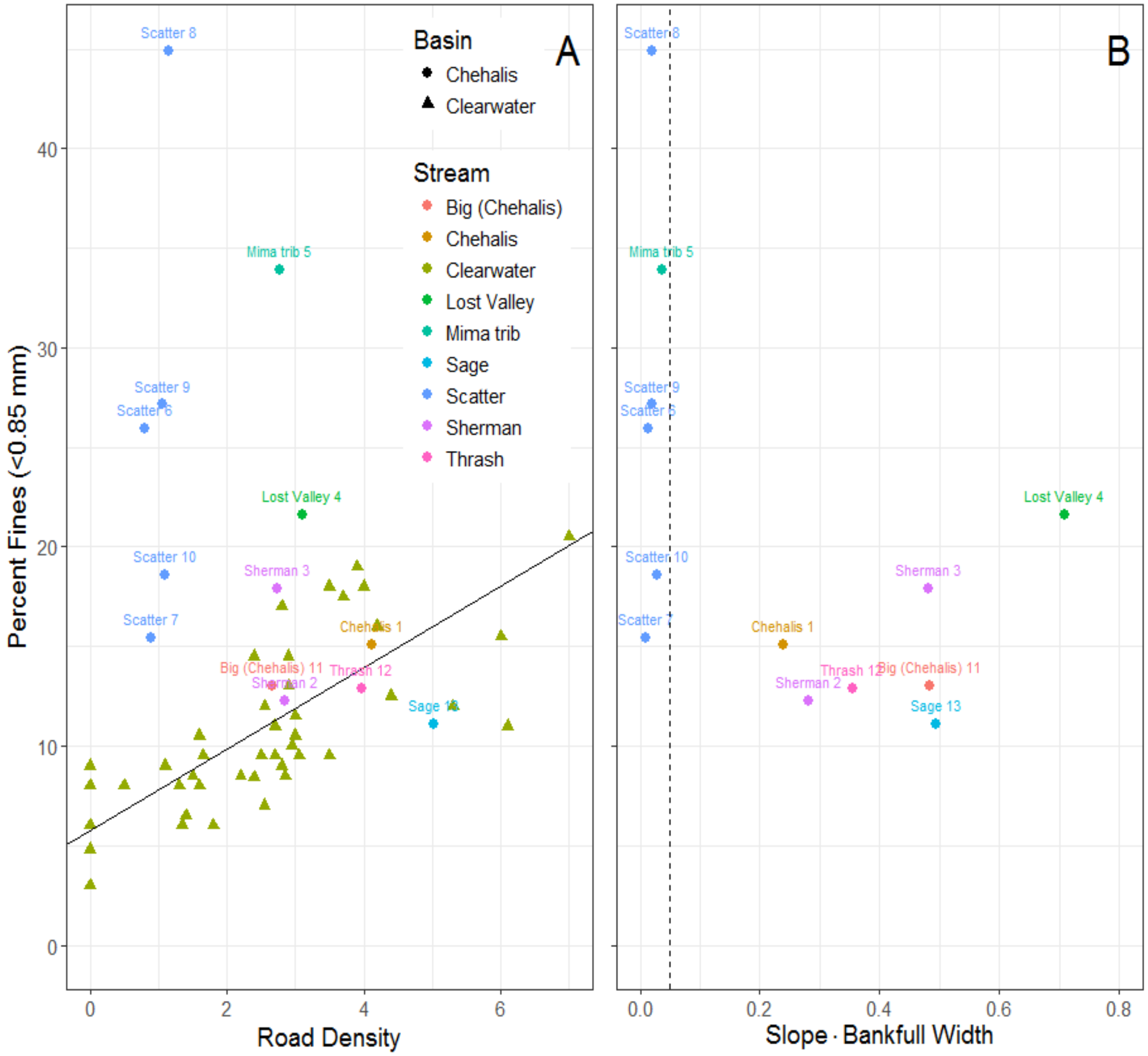

References

1. Mobrand Biometrics, Inc. Assessment of salmon and steelhead performance in the Chehalis River basin in relation to habitat conditions and strategic priorities for conservation and recovery actions. Vashon, WA: Mobrand Biometrics, Inc.; 2003.

2. Cederholm CJ, Reid LM, Edie BG, Salo EO. Effects of forest road erosion on salmonid spawning gravel composition and populations of the Clearwater River, Washington. In: Hashagen KH, editor. Habitat disturbance and recovery: proceedings of a symposium conducted January 1981. San Francisco, CA: California Trout, Inc.; 1982. pp. 1–17.
