# Supplementary figures and images for: A process-based assessment of landscape change and salmon habitat losses in the Chehalis River basin, USA
Source: PLoS One. 2021 Nov 2;16(11):e0258251. doi: 10.1371/journal.pone.0258251 (PMC8562855; doi:10.1371/journal.pone.0258251)

**S8 Figure. Map of historical floodplain habitats including marshes, ponds, and lakes.**

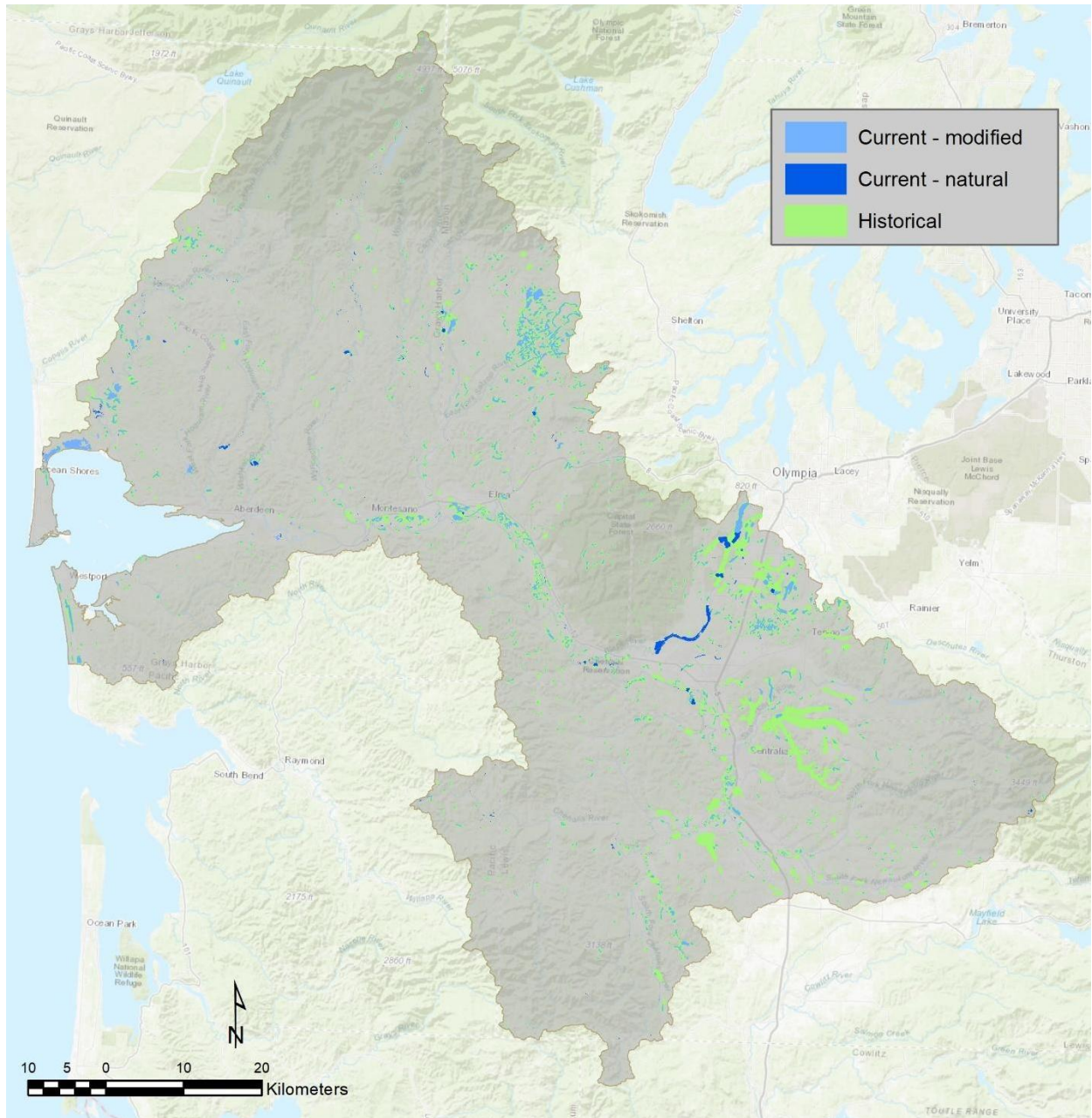

Supplement: S8 Fig — (PDF) [file pone.0258251.s008.pdf]
