# Supplementary material for: A process-based assessment of landscape change and salmon habitat losses in the Chehalis River basin, USA
Source: PLoS One. 2021 Nov 2;16(11):e0258251. doi: 10.1371/journal.pone.0258251 (PMC8562855; doi:10.1371/journal.pone.0258251)
Supplement: S1 Table — Table shows all values for the mainstem Chehalis and Chehalis tributaries by river reach. We developed historical length multipliers for each reach based on unpublished lidar-derived data from Natural Systems Design (NSD) and reference values of sinuosity and side-channel length [1, 2]. (PDF) [file pone.0258251.s009.pdf]

S1 Table. Large river main channel and side channel length multipliers. Table shows all values for the mainstem Chehalis and Chehalis tributaries by river reach. We developed historical length multipliers for each reach based on unpublished lidar-derived data from Natural Systems Design (NSD) and reference values of sinuosity and side-channel length [1,2].

River	Reach	Main Channel Multiplier	Side-Channel Multiplier
WF Satsop (1-5)	Middle Satsop Rd to D3000 Rd	1.3	1.2
WF Satsop (6,7)	D3000 Rd to Muller Rd	1	0.1
WF Satsop (8A)	Muller Rd to Cougar Smith Rd	1	1.5
WF Satsop (8B, 9)	Cougar Smith Rd up to lidar boundary	1	0.1
WF Satsop (11,12,13)	From beginning of lidar in Olympic foothills to FS Road 2153	1	0.6
EF Satsop (5-9)	South end Meadowlark Rd to forks	1.3	1.2
EF Satsop (10A,10B, 11,12A,12B,13,14A)	South end Meadowlark Rd to forks	1.3	1.2
EF Satsop (14B, 14C, 14D, 14E, 15)	W Plug Mill Rd to hatchery	1.1	1.1
Wishkah (5-7)	South end Riverside Rd to W Wishkah Rd	1.2	1.3
Wynoochee (2-15)	Olympic Hwy to N. End Matzen Rd	1.2	1.5
Humptulips (6A-15)	Robinson Rd to Big Creek	1.2	1.5
Humptulips (16-23B)	Big Creek to W Fork	1.2	1.5
Chehalis (54-62)	South Fork to Newaukum	1.1	0.7
Chehalis (49-53)	Newaukum to Skookumchuck	1.1	0.1
Chehalis (40-48)	Skookumchuck to Black	1.3	1
Chehalis (18-39)	Black to Satsop	1.1	1
Skookumchuck (1-9)	Mainstem	1.3	1.2

References

1. Beechie TJ, Liermann M, Pollock MM, Baker S, Davies J. Channel pattern and river-floodplain dynamics in forested mountain river systems. Geomorphology. 2006;78: 124–141. doi:10.1016/j.geomorph.2006.01.030

2. Collins BD, Montgomery DR. The legacy of Pleistocene glaciation and the organization of lowland alluvial process domains in the Puget Sound region. Geomorphology. 2011;126: 174–185. doi:10.1016/j.geomorph.2010.11.002
