# Supplementary material for: A process-based assessment of landscape change and salmon habitat losses in the Chehalis River basin, USA
Source: PLoS One. 2021 Nov 2;16(11):e0258251. doi: 10.1371/journal.pone.0258251 (PMC8562855; doi:10.1371/journal.pone.0258251)
Supplement: S2 Table — The ‘Reference’ column shows mean percent pool for the historical period, whereas land cover classes show current mean percent pool for each land cover and slope class. (PDF) [file pone.0258251.s010.pdf]

S2 Table. Mean percent pool area by slope class and land cover class. The ‘Reference’ column shows mean percent pool for the historical period, whereas land cover classes show current mean percent pool for each land cover and slope class.

Slope class	Percent pool by land cover class					
	**Reference**	**Forest**	**Wetland**	**Agriculture**	**Developed**	**Bare**
0-0.02	79%[a]	75%	89%	92%	74%	83%
0.02-0.04	66%[b]	48%	53%	60%	51%	50%
>0.04	35%[c]	34%	32%[d]	31%	35%[d]	35%

a. Updated based on higher proportion of very low gradient streams in the Chehalis basin
b. Based on reference sites [1,2]
c. Based on reference sites [3]
d. Value from the Bare category used as surrogate

References

1. Beechie T, Beamer E, Wasserman L. Estimating coho salmon rearing habitat and smolt production losses in a large river basin, and implications for habitat restoration. North American Journal of Fisheries Management. 1994;14: 797–811. doi:10.1577/1548-8675(1994)014<0797:ECSRHA>2.3.CO;2

2. Beechie TJ, Sibley TH. Relationships between channel characteristics, woody debris, and fish habitat in northwestern Washington streams. Transactions of the American Fisheries Society. 1997;126: 217–229. doi:10.1577/1548-8659(1997)126<0217:RBCCWD>2.3.CO;2

3. Beechie TJ, Collins BD, Pess GR. Holocene and recent geomorphic processes, land use, and salmonid habitat in two north Puget Sound river basins. In: Dorava JM, Montgomery DR, Palcsak BB, Fitzpatrick FA, editors. Geomorphic Processes and Riverine Habitat. Washington, D. C.: American Geophysical Union; 2001. pp. 37–54.
