# Supplementary material for: A process-based assessment of landscape change and salmon habitat losses in the Chehalis River basin, USA
Source: PLoS One. 2021 Nov 2;16(11):e0258251. doi: 10.1371/journal.pone.0258251 (PMC8562855; doi:10.1371/journal.pone.0258251)
Supplement: S3 Table — Estimated pond densities from maps and data showing calculation of weighted mean natural potential beaver ponds per km in the Chehalis basin [1]. Number of ponds is the manually-counted number of dam symbols in each subbasin from the published maps. (PDF) [file pone.0258251.s011.pdf]

**S3 Table. Beaver pond densities.** Estimated pond densities from maps and data showing calculation of weighted mean natural potential beaver ponds per km in the Chehalis basin [1]. Number of ponds is the manually-counted number of dam symbols in each subbasin from the published maps.

| Subbasin (as delineated in Wampler et al. 1993) | # Ponds | Stream Length (mi) | Stream Length (km) | Density (ponds/km) |
|---|---|---|---|---|
| Newman | 76 | 94 | 151 | 0.5 |
| Porter | 4 | 27 | 43 | 0.1 |
| Gibson | 19 | 38 | 61 | 0.3 |
| Scatter | 30 | 31 | 50 | 0.6 |
| China | 35 | 37 | 60 | 0.6 |
| Stearns | 35 | 20 | 32 | 1.1 |
| Elk | 39 | 43 | 69 | 0.6 |
| Lincoln | 111 | 63 | 101 | 1.1 |
| **Weighted mean** | | | | **0.6** |

References

1. Wampler PL, Knudsen EE, Hudson M, Young TA. Chehalis River basin fishery resources: Salmon and steelhead stream habitat degradations. Olympia, WA: U.S. Fish and Wildlife Service; 1993.
