# Supplementary material for: A process-based assessment of landscape change and salmon habitat losses in the Chehalis River basin, USA
Source: PLoS One. 2021 Nov 2;16(11):e0258251. doi: 10.1371/journal.pone.0258251 (PMC8562855; doi:10.1371/journal.pone.0258251)
Supplement: Attachment — Submitted filename: Response to Reviewers.docx [file pone.0258251.s012.docx]

8 July 2021

Tim Beechie

Northwest Fisheries Science Center

National Marine Fisheries Service

National Oceanic and Atmospheric Administration

2725 Montlake Blvd E.

Seattle, WA 98112

206.860.3409

tim.beechie@noaa.gov

RE: PONE-D-20-37830

We have addressed each of the editor reviewer comments as described in our detailed responses below. Our response are in *italic*. Line number locations for each comment refer to line numbers in the original manuscript that was reviewed. In our responses we describe the location of revisions with line numbering from the revised manuscript for easier location of the revisions.

**Editor comments**

1. We rechecked the reference list to assure that it is complete. We added a citation for Jorgensen et al. in review that can be modified once both manuscripts are accepted.

2. We have followed the file naming convention for PLOS ONE, submitting three files: Response to Reviewers, Revised Manuscript with Track Changes, and Manuscript.

3. We would like to be considered for the Freshwater Ecosystems Call for Papers.

*Thank you.*

4. Competing interests.

i. Revised funding statement

*The funders provided support in the form of salaries for authors TB, CF, CN, BT-P, and one of the funders (Washington Department of Fish and Wildlife) provided several data sets. The funders did not have any additional role in the study design, data collection and analysis, decision to publish, or preparation of the manuscript. Authors affiliated with Ocean Associates, Inc. and A.I.S., Inc. were under contract to the National Marine Fisheries Service and funded under the same contracts; these commercial companies provided no funding for the project. The specific roles of these authors are articulated in the ‘author contributions’ section.*

ii. Revised competing interests statement

*The authors have declared that no competing interests exist. Authors affiliated with Ocean Associates, Inc. and A.I.S., Inc. were under contract to the National Marine Fisheries Service. This does not alter our adherence to PLOS ONE policies on sharing data and materials.*

5. Data sets and model code are available at: <https://www.fisheries.noaa.gov/resource/tool-app/habitat-assessment-and-restoration-planning-harp-model>.

6. We have added the captions for Supporting Information at the end of the manuscript.

7. S2 Figure contained background imagery from Google Earth. Because that figure was not critical to the methods, we modified the methods description in the text and deleted that figure.

**Reviewer comments**

77: Authors do a nice job placing the objectives of this paper in the context of the 3-step approach.

*Thank you.*

85: If the restoration is process-based, why is the measure of impact pattern based?

*The modeling sequence requires that we document changes in habitat conditions so that habitat changes can be translated into life-stage parameters for the life cycle models. The habitat changes result from changes in processes (e.g., increased fine sediment is a result of road surface erosion), and restoration actions can focus on process restoration (reducing road surface erosion), but the ultimate outcome is a change in habitat conditions (fine sediment in spawning gravel). We did not add text as this is noted in lines 54-56, and in 105-111.*

88: How about natural conditions varying temporally, given giant variation in salmon production prior to contemporary land use (e.g., Rogers et al. PNAS 2013 Centennial-scale fluctuations)? Could help to briefly address temporal variation when introducing spatial variation, so it doesn’t come off as assuming that the past was static.

*We added text to address this point (line 191-194), and explain why we do not model temporal variation in these papers. We also note that subsequent manuscripts will include climate change and stochastic variation in stream flow and temperature.*

90: could end with ‘,respectively’ I had to read this twice to understand this sentence.

*Adding ‘respectively’ seemed to slightly alter the meaning of the sentence. We rewrote it as “The second step is translating habitat conditions into life-stage capacities and productivities for each species and habitat scenario, where habitat scenarios represent potential habitat improvements from alternative restoration actions.” Lines 88-91.*

99: Does ‘dial’ imply that they can be manipulated by management?

*Yes. To clarify we added text at the end of the sentence: “…to represent management options that can restore processes and improve habitat conditions.” We also revised text in lines 105-111 and Figure 1 so that the ‘dials’ are called restoration actions instead of drivers.*

134: Is there typically enough data to quantify population bottlenecks in terms of the habitat features being restored? For example, if marshes provided flood refuge or spring foraging, when is there the data to understand how area of marsh and fish density translate into survival? Isn’t the resolution of data more coarse than that needed to address the processes and functions being restored? [OK I see that the life-cycle paper is separate from this one, so this comment doesn’t need to be addressed in this manuscript].

*Yes. This is addressed in the life-cycle model paper.*

221: Many readers may wonder how climate change fits in with this and how we assess baseline conditions if they unlikely to occur in future climates.

*Agreed. We address this in the discussion starting at line 846, and also in 3 other manuscripts that will build upon these two manuscripts to address climate change effects on stream flow and temperature.*

Fig. 1 Why is floodplain connectivity a driver? Isn’t this a habitat condition? The Driver/Habitat conditions differentiation isn’t that clear for me, so it could be explained a little more when introduced…. Or the terms could be reworded to sound like drivers. For example floodplain connectivity (a habitat condition in my mind) could be rephrased so that it was parallel with terms such as road density and channel straightening. But in general the mix up between mechanisms and patterns here is confusing.

*We revised the manuscript so that all but one use of the word ‘driver’ was changed to ‘restoration action’ to simplify the text, including in Figure 1. We also changed some of the action names in Fig 1 to better reflect a restoration action.*
